# Spatial Change of Cruciate Ligaments in Rat Embryo Knee Joint by Three-Dimensional Reconstruction

**DOI:** 10.1371/journal.pone.0131092

**Published:** 2015-06-22

**Authors:** Xiangkai Zhang, Tomoki Aoyama, Ryota Takaishi, Shinya Higuchi, Shigehito Yamada, Hiroshi Kuroki, Tetsuya Takakuwa

**Affiliations:** 1 Human Health Science, Graduate School of Medicine, Kyoto University, Kyoto, Japan; 2 Congenital Anomaly Research Center, Graduate School of Medicine, Kyoto University, Kyoto, Japan; Université de Lyon—Université Jean Monnet, FRANCE

## Abstract

This study aimed to analyze the spatial developmental changes of rat cruciate ligaments by three-dimensional (3D) reconstruction using episcopic fluorescence image capture (EFIC). Cruciate ligaments of Wister rat embryos between embryonic day (E) 16 and E20 were analyzed. Samples were sectioned and visualized using EFIC. 3D reconstructions were generated using Amira software. The length of the cruciate ligaments, distances between attachment points to femur and tibia, angles of the cruciate ligaments and the cross angle of the cruciate ligaments were measured. The shape of cruciate ligaments was clearly visible at E17. The lengths of the anterior cruciate ligament (ACL) and posterior cruciate ligament (PCL) increased gradually from E17 to E19 and drastically at E20. Distances between attachment points to the femur and tibia gradually increased. The ACL angle and PCL angle gradually decreased. The cross angle of the cruciate ligaments changed in three planes. The primordium of the 3D structure of rat cruciate ligaments was constructed from the early stage, with the completion of the development of the structures occurring just before birth.

## Introduction

The cruciate ligaments of the knee joint are a pair of ligaments arranged in the shape of an X.[1] The cruciate ligaments consist of the anterior cruciate ligament (ACL) and the posterior cruciate ligament (PCL). The ACL lies anterolaterally, connecting the anterior part of the upper surface of the tibia to the inner aspect of the lateral condyle of the femur.[2] The PCL lies posteromedially and attaches the back of the upper surface of the tibia to the inner aspect of the medial condyle of the femur.[3] They are important for stabilizing the articulating bones (femur and tibia), especially during movement.[4] The ACL resists anterior instability and internal rotation of the tibia.[5] The PCL resists posterior instability.[3] Injury of the cruciate ligaments can lead to knee instability, meniscal damage, and osteoarthritis (OA). Because of the importance of cruciate ligament function, the ACL transection and reconstruction rat model has been widely used. Knowledge of the three dimensional (3D) anatomic structure and biomechanics of the cruciate ligaments is important for understanding the biology and the clinical significance of the cruciate ligaments.

The development of the cruciate ligaments is indispensable to the formation of the knee joint. In the human embryo, the chondrification of the femur and tibia begins at Carnegie stage (CS) 18. At CS 21, the PCL is distinguishable. At CS 23, the ACL and PCL are clearly visible and joint cavity formation is recognized.[6] Although the development of the cruciate ligaments has been investigated histologically, there are few reports about the anatomical development of the cruciate ligaments in 3D. In our previous study reported by Takaishi,[7] we showed the 3D structural development of the rat knee joint using episcopic fluorescence image capture (EFIC). EFIC creates data on volumes and coordinates by imaging with tissue autofluorescence. After capturing the image, the continuous sections were used to create 3D computer models.[8] Using this technique,[9] it is possible to precisely analyze the spatial variation of the embryo during development. In Takaishi’s report, we analyzed the development of the joint cavity. We succeeded in describing the spatial changes involved in cavity formation. In the current study, the development of the cruciate ligaments of the rat knee joint was investigated in 3D using EFIC.

## Materials and Methods

### Animals

Eighteen right hindlimbs were removed from 18 white Wister rat embryos between E16 and E20 (E16, n = 2; E17, 18, 19, 20, n = 4 each). Wister rats were sourced from SHIMIZU Laboratory Supplies Co., Ltd (Kyoto, Japan).The mother rats were euthanized by pentobarbital sodium overdose before caesarean section. The rat embryos were fixed whole immediately after removal from the uterus in 4% paraformaldehyde at 4°C overnight before dissecting the hindlimbs.

### Preparation and workflow for EFIC

Preparation of samples for EFIC was performed as described in our previous study.[7] Briefly, for EFIC, the dehydrated samples were infiltrated and embedded in 70.4% paraffin wax containing 24.9% Vyber, 4.4% stearic acid, and 0.4% sudan IV.[10] The paraffin blocks were sectioned using a Leica SM2500 sliding microtome (Leica Microsystems, Bannockburn, UK) at 6–10 μm. Autofluorescence at the paraffin block face was visualized using epifluorescence imaging with mercury illumination and a discosoma Redfilter. Fluorescent images were captured using a Hamamatsu ORCA-ER low-light CCD camera (HAMATSU Photonics K.K., Shizuoka, Japan). After capturing an image of the block face, a small slice of the block was removed using the microtome blade. This slice permitted preservation of histologic sections for H&E staining. Then, a digital image of the freshly cut block surface was captured and the next slice of embedding block was removed. This procedure was repeated until the region of interest was sectioned and a stack of aligned digital images showing subsequent block faces with tissues of the specimens was produced.

### 3D reconstruction and coordinate location

Serial-section images of rat embryos were obtained. The femur, cruciate ligament, and tibia were outlined in three different colors by different autofluorescence intensity between cruciate ligament and bone. Cruciate ligament showed relatively high autofluorescent intensity due to dense distribution of cells, whereas the bones (femur and tibia), which were chondrifying, showed relatively low intensity. The interested parts, which were outlined manually, were reconstructed three dimensionally without smoothing (S1 Video) using AMIRA 5.4.3 software (Visage, Berlin, Germany).

All coordinates of the 3D reconstruction model were generated automatically after 3D reconstruction was completed. The coordinates of the attachment points of the cruciate ligament were outlined manually and generated automatically using the barycentric coordinates of the contact area of bone and ligament. The coordinates of the cross point of the cruciate ligaments were outlined manually and generated automatically using the barycentric coordinates of the cruciate ligament intersection area.

### Analysis

**Fig 1:**
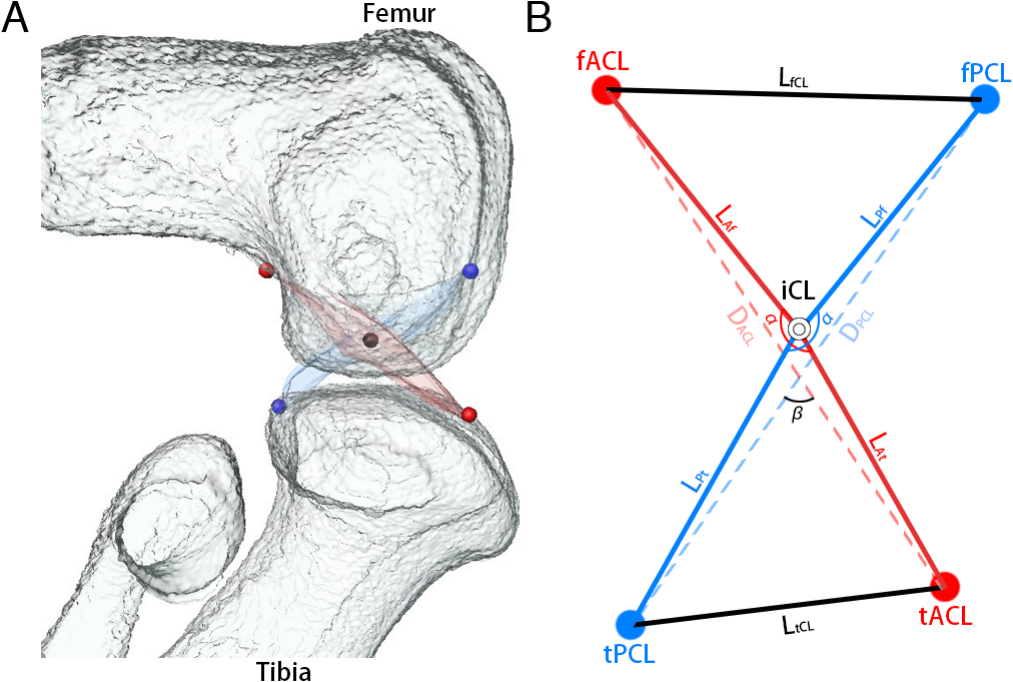
Imaging of measurement points. A: A 3D reconstruction of the knee joint was generated from 2D continuous sections with Amira software. Femur and tibia (white), ACL (red) and PCL (blue). Red points indicate the attachment of the ACL to the femur and tibia. Blue points indicate the attachment of the PCL to the femur and tibia. The black point indicates the cross point of both ligaments. B: Measurement points. fACL: femoral attachment of ACL. fPCL: femoral attachment of PCL. tACL: tibial attachment of ACL. tPCL: tibial attachment of PCL. The cross point of ACL and PCL was indicated as intersection of cruciate ligament (iCL). Length of ACL (L_ACL_) was calculated by length of ACL on femoral side (L_Af_) plus length of ACL on tibial side (L_At_). Length of PCL (L_PCL_) was calculated by length of PCL on femoral side (L_Pf_) plus length of PCL on tibial side (L_Pt_).

The cross point of the cruciate ligaments was designated as “Intersection of Cruciate Ligaments” (iCL). The attachment points of the cruciate ligaments to the femur and tibia were designated as “femoral attachment of ACL” (fACL), “femoral attachment of PCL” (fPCL), “tibial attachment of ACL” (tACL), and “tibial attachment of PCL” (tPCL) (Fig 1). These five coordinates were used to calculate lengths and angles.

The length of the ACL was calculated as the sum of the length of the ACL on the femoral side (L_Af_) and the length of the ACL on the tibial side (L_At_). The length of the PCL was calculated as the sum of the length of the PCL on the femoral side (L_Pf_) and the length of the PCL on the tibial side (L_Pt_). The distance between fACL and fPCL was defined as L_fCL_. The distance between tPCL and tACL was defined as L_tCL_. The ACL angle (αACL) was measured at the angle between fACL, iCL and tACL. The PCL angle (αPCL) was measured at the angle between fPCL, iCL and tPCL. The distance between the fACL and tACL was defined as D_ACL_. The distance between the fPCL and tPCL was defined as D_PCL_. The cross angle (β) of the cruciate ligaments was measured at the angle between D_ACL_ and D_PCL_ in the sagittal, frontal, and horizontal planes [β(sagittal)/β(frontal)/β(horizontal)] (Fig 1B).

To learn more about the spatial change of cruciate ligament three dimensionally, the mean coordinates of attachment of the cruciate ligament at each stage were integrated into the same coordinate system. A standard position, which was required, was obtained as follows using Matlab 2014a software (MathWorks, Natick, MA). (1) iCL was the original point. (2) the femoral condylar axis was parallel to the X axis. (3) fACL and fPCL were above the X axis, while tACL and tPCL were below the X axis.

### Statistical analysis

All data are shown as actual values. SPSS software (IBM, Armonk, NK) was used for statistical analysis. To examine differences between stages, one-way analysis of variance (ANOVA) followed by the Tukey-Kramer or Dunnett T3 test was used.

### Ethics

All of the experiments with animals were approved by the Institutional Animal Research Committee and performed according to the Guidelines for Animal Experiments of Kyoto University (Permit Number: 14038). Care of the animals was in accordance with the Kyoto University guidelines.

## Results

### Hematoxylin Eosin (HE) staining of cruciate ligaments

**Fig 2:**
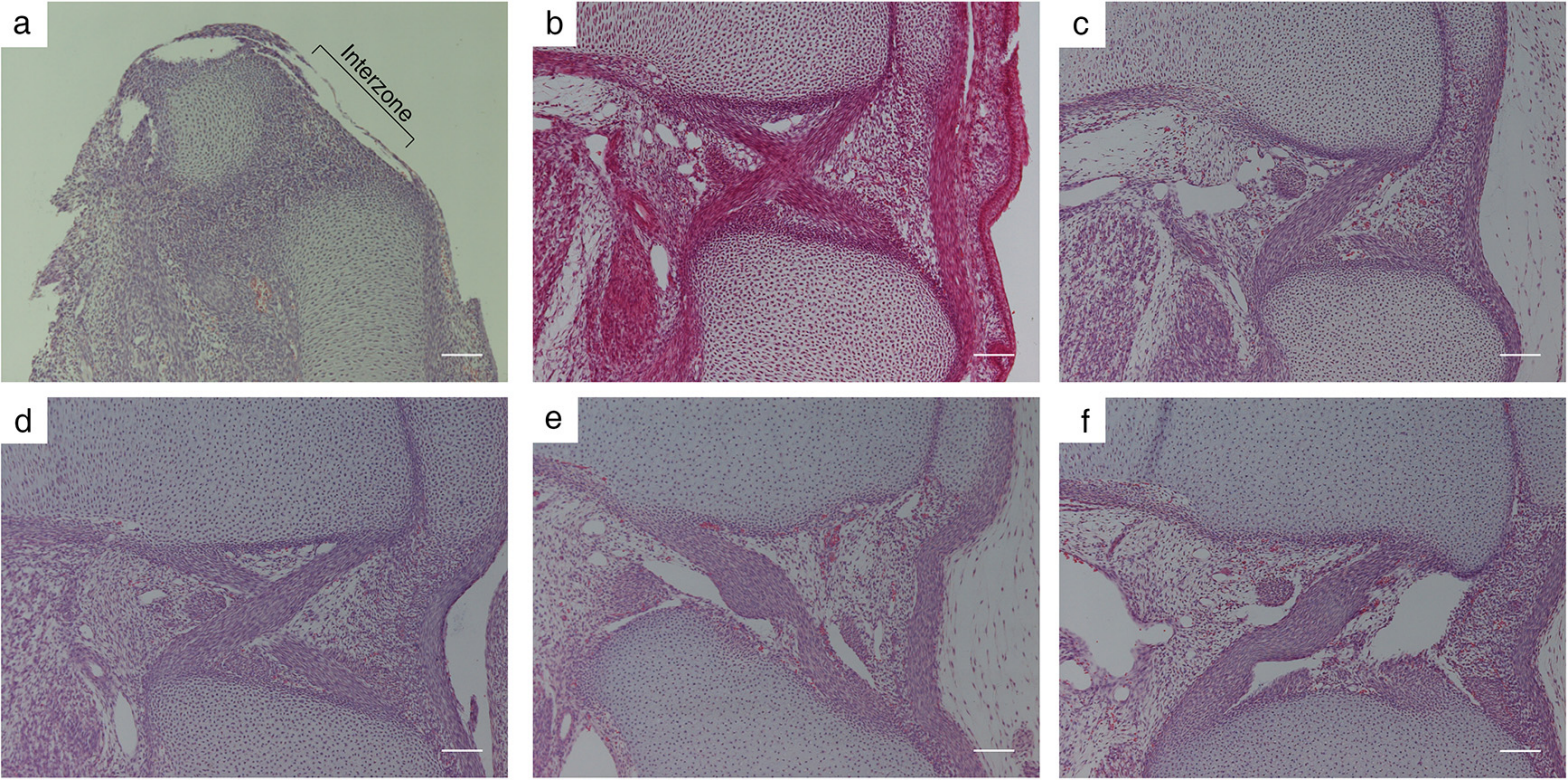
Histological findings of cruciate ligaments. Histological images of sagittal sections of the knee joint by HE staining. a: Embryonic day (E) 16. The cross section of the knee showed no signs of the ACL and PCL in the interzone. b: E17. The cross section of knee clearly showed the ACL and PCL. c: E18. The cross section showed the PCL. d: E19. The cross section showed the ACL and PCL. e: E20. The border and bundle of the ACL were clearly visible. f: E20. The cross sections of the knee showed buckling of the ACL and PCL. Magnification x100. Bar = 100 μm.

The knee joint was observed using histological sections with HE staining between E16 and E20 (Fig 2). A low-density area corresponding to chondrification was seen between the femur, tibia, and fibula in histological section at E16 (Fig 2A). A three-layered structure corresponding to the interzone was seen as an area of higher cell density between the femur and tibia. The borders between the interzone and the bone primordia were not distinct. The ACL, PCL and cavity could not be observed at E16. The ACL and PCL were detected as a condensed group of spindle cells between the femur and tibia at E17 (Fig 2B). Loose mesenchymal cells with small capillaries containing erythrocytes and small cavities with thin epithelial cell walls were present around the ligaments. The bone primordia become distinct as the border is lined with the single layered cells. The direction of fiber growth could be distinguished at E18 (Fig 2C), and a clear difference in direction was observed at E19 (Fig 2D). The borders of the ligaments become sharp as the synovial cavity becomes large and close to the ligaments at E20 (Fig 2E and 2F). The ligaments seemed not to run straight and to be irregular in diameter in 2D sections, because of the buckling of the cruciate ligaments. The attachments of the ligaments were broad in width.

### Length of cruciate ligaments

**Fig 3:**
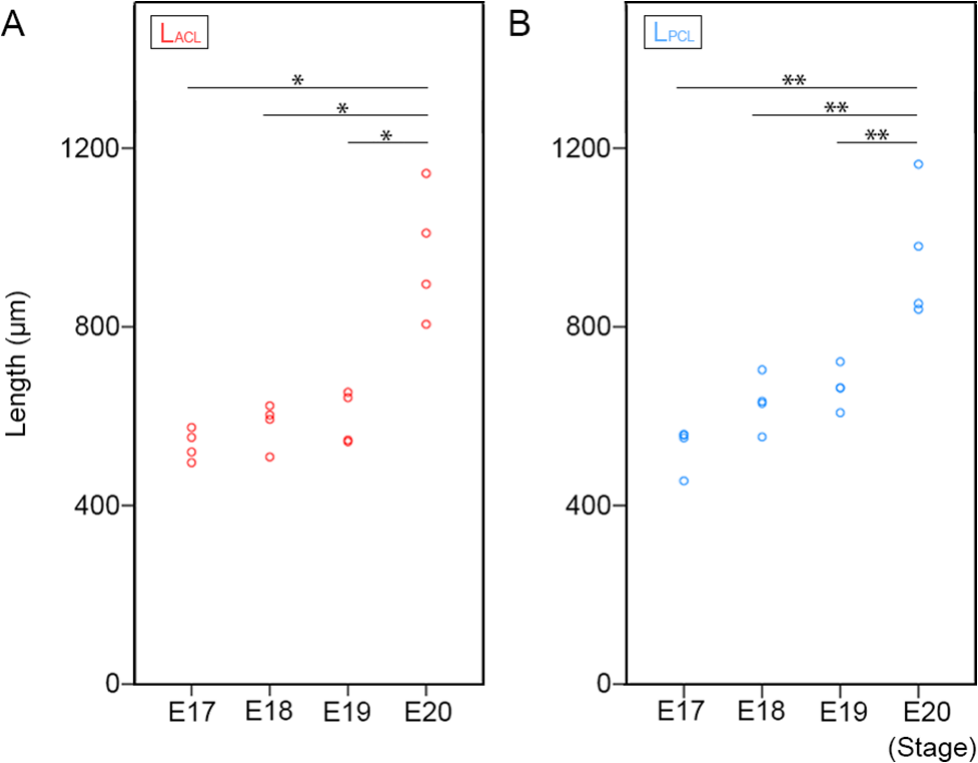
Length of cruciate ligaments. A: The length of the ACL at E17, E18, E19, and E20. The small red circles indicate the length of the ACL in each sample. *p < 0.05 (Dunnett T3 test). B: The length of the PCL at E17, E18, E19, and E20. The small blue circles indicate the length of the PCL in each sample. ** *p* < 0.01 (Tukey-Kramer test).

The lengths of the cruciate ligaments were measured as L_ACL_ and L_PCL_ (Fig 1) at each stage. Because the borders of the cruciate ligaments became clear at E17, the lengths of them were measured from E17 (Fig 2B). The mean L_ACL_ gradually increased, but not significantly, from E17 to E19 (E17: 535.3 ± 39.3 μm, E18: 566.0 ± 57.3 μm, E19: 598.1 ± 55.3 μm), however drastically increased with a significant difference at E20 (913.6 ± 299.9 μm) (Fig 3A).

The mean L_PCL_ gradually increased from E17 to E19 (E17: 506.1 ± 52.0 μm, E18: 627.3 ± 75.0 μm, E19: 663.4 ± 57.0 μm), and drastically increased with a significant difference at E20 (999.8 ± 162.1 μm) (Fig 3B).

### Distance between attachments of cruciate ligaments

**Fig 4:**
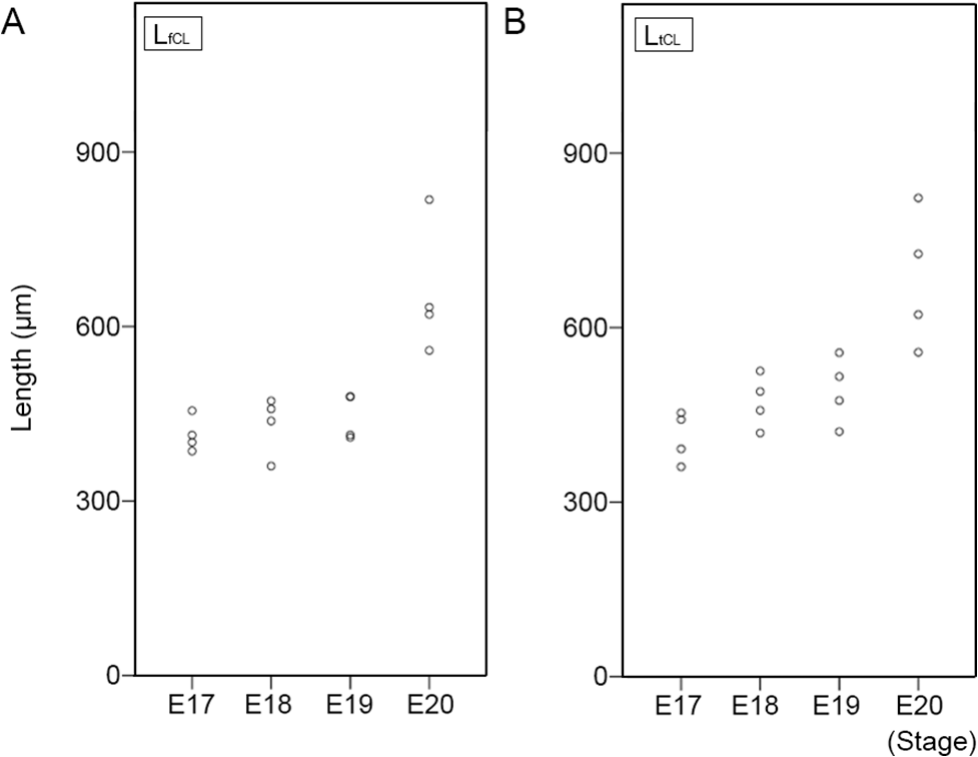
The distance between the attachment points of the cruciate ligaments on the femur and tibia. A: The length of the distance between the attachment points of the ACL and PCL on the femur (L_fCL_). The small black circles indicate the distance in each sample. *p* < 0.05 (Test of Homogeneity of Variances). B: The length of the distance between the attachment points of the ACL and PCL on the tibia (L_tCL_). The small black circles indicate the distance in each sample. *p* < 0.05 (Test of Homogeneity of Variances).

The distances between the attachments of the cruciate ligaments at the femur and tibia were measured as L_fCL_ and L_tCL_ respectively (Fig 1) at each stage. The mean L_fCL_ gradually increased, but not significantly, from E17 to E20 (E17: 420.7 ± 34.8 μm, E18: 432.1 ± 40.1 μm, E19: 444.6 ± 35.2 μm, E20: 612.3 ± 205.9 μm) (Fig 4A).

The mean L_tCL_ gradually increased, but not significantly, from E17 to E20 (E17: 408.2 ± 46.5 μm, E18: 473.2 ± 53.3 μm, E19: 490.2 ± 68.0 μm, E20: 691.7 ± 132.8 μm) (Fig 4B).

### Angle of cruciate ligaments

**Fig 5:**
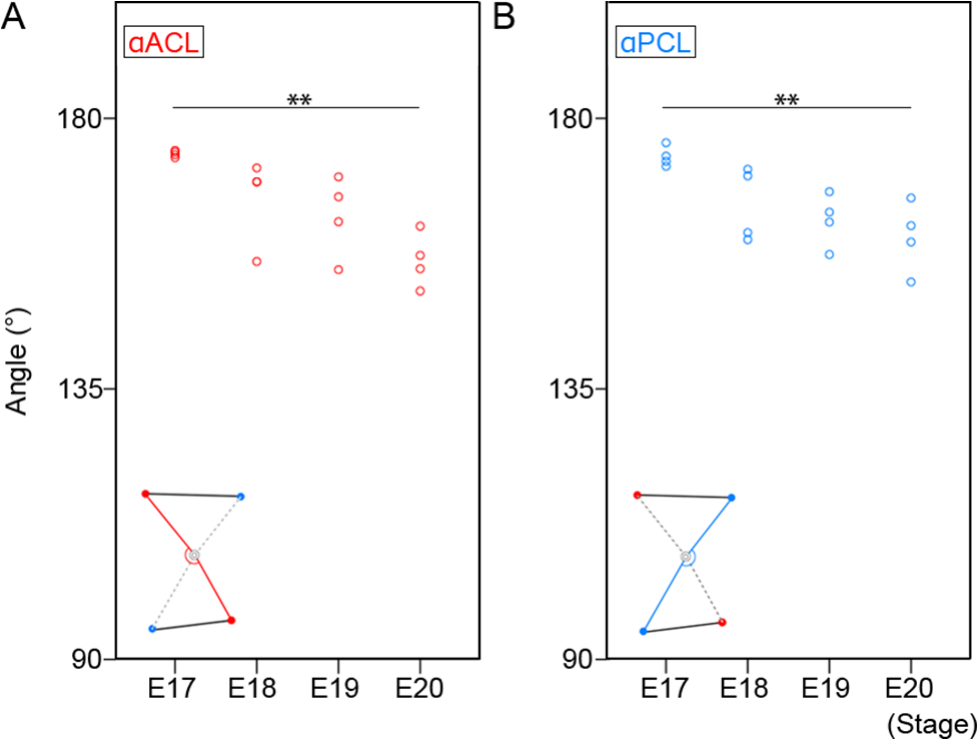
Angle of cruciate ligaments. A: The angle of ACL (αACL) at E17, E18, E19, and E20. The small red circles indicate the angle of ACL in each sample. ** *p* < 0.01 (Tukey-Kramer test). B: The angle of PCL (αPCL) at E17, E18, E19, and E20. The small blue circles indicate the angle of PCL in each sample. ** *p* < 0.01(Tukey-Kramer test).

The cruciate ligaments are not completely straight, but slightly curved.[11] The curves of the ligaments were measured as ACL angle (αACL) and PCL angle (αPCL) (Fig 5). The mean αACL slowly decreased from E17 to E19 (E17: 174.1 ± 0.6°, E18: 164.0 ± 7.8°, E19: 162.6 ± 7.7°) and significantly decreased at E20 (156.7 ± 5.4°) (Fig 5A).

The mean αPCL slowly decreased from E17 to E19 (E17: 174.1 ± 2.0°, E18: 165.8 ± 5.3°, E19: 162.7 ± 5.2°) and significantly decreased at E20 (159.9 ± 7.0°) (Fig 5B).

### Cross angle between cruciate ligaments

**Fig 6:**
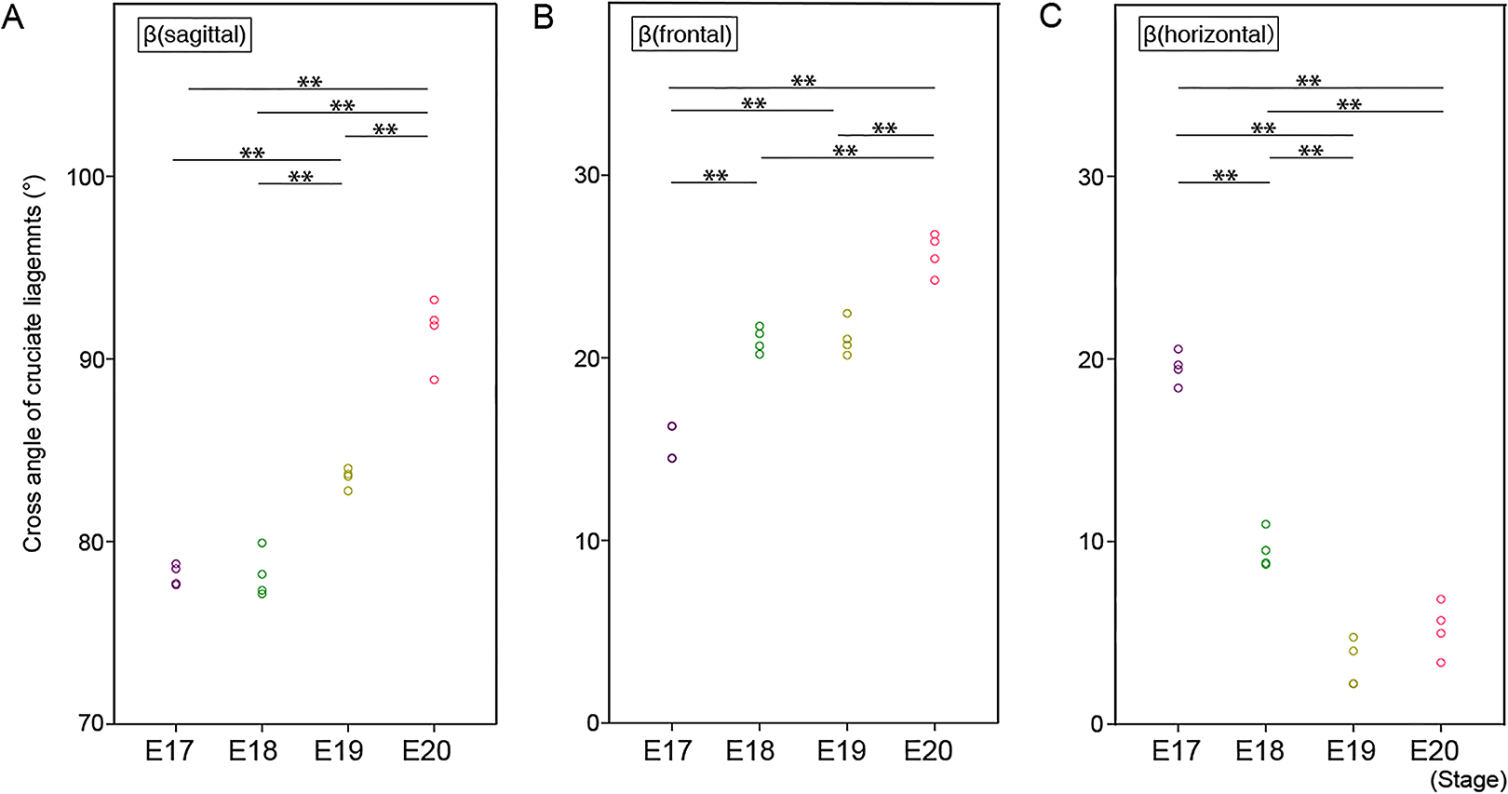
Cross angle of cruciate ligaments in each dimension. A: Cross angle of cruciate ligaments in the sagittal plane [β(sagittal)]. Each small circle indicates the cross angle in each sample (purple; E17, green; E18, yellow; E19, red; E20). ** *p* < 0.01 (Tukey-Kramer test). B: Cross angle of cruciate ligaments in the coronal plane [β(frontal)]. Each small circle indicates the cross angle in each sample (purple; E17, green; E18, yellow; E19, red; E20). ** *p* < 0.01 (Tukey-Kramer test). C: Cross angle of cruciate ligaments in the horizontal plane [β(horizontal)]. Each small circle indicates the cross angle in each sample (purple; E17, green; E18, yellow; E19, red; E20). ** *p* < 0.01 (Tukey-Kramer test).

The cruciate ligaments cross each other in three dimensions. The cross angle of the cruciate ligaments was measured in the sagittal, coronal, and horizontal planes [β(sagittal)/β(frontal)/β(horizontal)]. The mean β(sagittal) significantly increased at E19 (E17: 78.2 ± 0.6°, E18: 78.5 ± 1.4°, E19: 83.4 ± 0.6°) and further increased at E20 (E20: 90.3 ± 1.5°) (Fig 6A). The mean β(frontal) significantly increased at E18 (E17: 15.4 ± 0.9°, E18: 21.0 ± 0.8°), then increased at E20 (E19: 21.3 ± 1.1°, E20: 25.5 ± 1.3°) (Fig 6B). The mean β(horizontal) decreased at E18 (E17: 19.5 ± 1.1°, E18: 9.8 ± 1.1°) and did not change obviously subsequently (E19: 3.5 ± 1.3°, E20: 5.1 ± 1.7°) (Fig 6C).

### Spatial change of attachment points of cruciate ligaments

**Fig 7:**
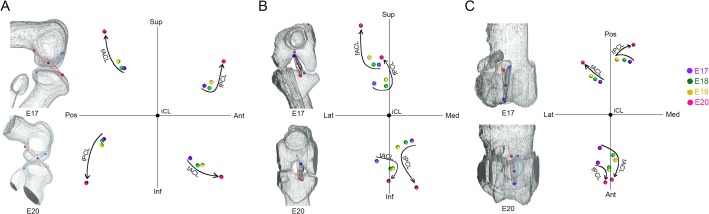
Spatial changes of the attachment points of the cruciate ligaments in each dimension. The average coordinates of the attachment points at each stage (purple; E17, green; E18, yellow; E19, red; E20) in the sagittal (A), frontal (B), and horizontal (C) planes. Femoral attachment of ACL (fACL). Femoral attachment of PCL (fPCL). Tibial attachment of ACL (tACL). Tibial attachment of PCL (tPCL). Superior (Sup), interior (Inf), anterior (Ant), posterior (Pos), medial (Med), lateral (Lat).

To understand the spatial changes of the cruciate ligaments, the mean coordinates of the four attachment points of the cruciate ligaments (fACL, fPCL, tACL, tPCL) were plotted at each stage in sagittal (Fig 7A), coronal (Fig 7B) and horizontal planes (Fig 7C).

In the sagittal plane, the four attachment points became separated in four directions (Fig 7A). The separation was gradual from E17 to E19, and drastic at E20 (Fig 7A).

In the frontal plane, the attachment points to the femur (fACL, fPCL) and tibia (tACL, tPCL) became separated in opposite directions in both cruciate ligaments (Fig 7B). The separation was gradual from E17 to E19, and drastic at E20 (Fig 7B). Femoral attachments (fACL, fPCL) displaced laterally and tibial attachments (tACL, tPCL) medially (Fig 7B).

In the horizontal plane, the posterior attachments (fACL, tPCL) spread out in a fan-like form, but the anterior attachments (fPCL, tACL) converged (Fig 7C). In this plane, the changes in position were more gradual.

## Discussion

In this study, the spatial changes of rat cruciate ligaments during development were analyzed using EFIC and 3D reconstruction. The cruciate ligaments were clearly observed at E17. The length of both ligaments increased, changing gradually from E17 to E19 and drastically at E20. The distance between the attachments of the ACL and PCL increased, changing gradually from E17 to E19 and drastically at E20. The mean αACL and αPCL gradually decreased. The change in β(sagittal) was significant at E20. The change in β(frontal) was gradual and the change in β(horizontal) was significant between E17 and E18.

These results suggest that in the development of the cruciate ligaments, distance changes are parallel, but angle changes are not typical. The elongation of the ligaments is in parallel with femoral and tibial bone development. The angle change is more complicated. The ACL lies in a front and lateral position, and PCL lies in a back and medial position. The three dimensional crossing structure of the cruciate ligaments is not just a simple cross, but also contains an element of torsion. The change of the cross angle is not parallel between the three planes (Fig 6). Current results may suggest that the torsional structure is constructed in a systematic manner.

The angle of the cruciate ligaments in humans is of great clinical significance. Both ligaments are not completely straight, but the buckling of the PCL is increased when the ACL is ruptured.[12, 13] The mean PCL angle is 123° in the normal human adult, but this decreases to 106° in the ACL-deficient knee.[14] The PCL angle itself changes with growth. The mean PCL angle in the child knee increases by a mean of 0.68° with each additional year of age.[11] The mean value of the PCL angle in open physes is 113.9° becoming 121.9° in closed physes.[11] These results suggest that the physiological buckling of the PCL changes according to growth. In the current study, the buckling of the cruciate ligaments was not significant in their formation stage (Fig 2B and 2C and Fig 5). The buckling of cruciate ligaments was gradually generated in later stages (Fig 2E and 2F and Fig 5). There is no information on the clinical significance of the evaluation of the buckling of the cruciate ligaments in rats, but this information on the active mechanism that forms the structure may contribute to the understanding of the physiology of the cruciate ligaments.

There seems to remain one question about the difference in timing of when the cruciate ligaments develop between human and rodents. The manner of the development of the knee joint itself is similar between the species.[15] The histological findings of the mesenchymal condensation, chondrification, the homogenous interzone, the separation of the femur, tibia and patella, ossification of the bones, and the cavitation of the joint are observed similarly in humans[16] and rats (Fig 2). The primordium of the cruciate ligaments are observed as cellular condensation at CS 19 in human [16, 17] and E17 in rats (Fig 2B). The joint formation is completed when the joint cavity is formed at CS 23 in humans[16] and E20 in rats.[7] Although the completion of cruciate ligaments’ formation is just before birth in rats, the development of the human embryo continues after the ligaments’ formation. In human knee development, the lower limbs became internally rotated from CS 19 to CS 23.[18] In rats, there is little information about the limb position in the fetal stage. A detailed analysis using 3 dimensional techniques in both species is needed to clear this issue, but the results from the current study will contribute to a better understanding of embryological features and functions of the cruciate ligaments.

## Conclusions

3D models of the structure of the cruciate ligaments at different stages in their development were successfully formed. The 3D crossing structure of the cruciate ligaments and buckling of the PCL were actively formed. The formation of ligaments was completed at the end of embryonic period. These findings will contribute to further understanding of cruciate ligaments function.

## Supporting Information

S1 Video 3D structure of knee and cruciate ligament of E17 rat. (MPG)
